# A novel highly pathogenic H5N8 avian influenza virus isolated from a wild duck in China

**DOI:** 10.1111/irv.12289

**Published:** 2014-11-01

**Authors:** Shengtao Fan, Lichen Zhou, Di Wu, Xiaolong Gao, Enle Pei, Tianhou Wang, Yuwei Gao, Xianzhu Xia

**Affiliations:** a Institute of Medical Biology, Chinese Academy of Medical Sciences & Peking Union Medical CollegeKunming, China; b Research Center of Wildlife Disease, Key Laboratory of Jilin Province for Zoonosis Prevention and Control, Military Veterinary Research Institute of Academy of Military Medical SciencesChangchun, China; c Laboratory of Wildlife Epidemic Diseases, East China Normal UniversityShanghai, China; d Shanghai Municipal Agency of Wildlife ConservationShanghai, China; e State Key Laboratory of Veterinary Biotechnology, Harbin Veterinary Research Institute, Chinese Academy of Agricultural SciencesHarbin, China

**Keywords:** H5N8, highly pathogenic avian influenza virus, wild duck

## Abstract

Migrating wild birds are considered natural reservoirs of influenza viruses and serve as a potential source of novel influenza strains in humans and livestock. During routine avian influenza surveillance conducted in eastern China, a novel H5N8 (SH-9) reassortant influenza virus was isolated from a mallard duck in China. blast analysis revealed that the HA, NA, PB1, PA, NP, and M segments of SH-9 were most closely related to the corresponding segments of A/duck/Jiangsu/k1203/2010 (H5N8). The SH-9 virus preferentially recognized avian-like influenza virus receptors and was highly pathogenic in mice. Our results suggest that wild birds could acquire the H5N8 virus from breeding ducks and spread the virus via migratory bird flyways.

*Please cite this paper as:* Fan *et al*. (2014) A novel highly pathogenic H5N8 avian influenza virus isolated from a wild duck in China. Influenza and Other Respiratory Viruses 8(6), 646–653.

## Introduction

Migrating wild birds are considered natural reservoirs of influenza viruses and have been implicated in the emergence and spread of novel influenza strains in humans and livestock.^1^,^2^ Since 1997, highly pathogenic avian influenza (HPAI) H5N1 viruses have caused numerous outbreaks in poultry in Asia and have sporadically caused human infections. H5N1 viruses are consistently found in different species of migratory birds along Eurasian flyways, leading to dissemination of H5N1 viruses and HPAI outbreaks in approximately 40 countries.^3^ The diversity of influenza viruses in avian species and viral transmission between domestic poultry and wild birds can lead to genomic reassortment events and the generation of novel influenza virus subtypes.^4^ In 2010, reassortment H5N8 and H5N5 viruses were found in breeding ducks in eastern China,^5^,^6^ although it is not yet clear whether these viruses are spread by wild birds as has been the case with H5N1 viruses.

During routine avian influenza surveillance conducted in eastern China, we isolated a novel H5N8 subtype virus from an apparently healthy mallard duck (*Anas platyrhynchos*). Although H5N8 avian influenza outbreaks have been reported the Republic of Korea (http://www.oie.int/en/animal-health-in-the-world/update-on-avian-influenza/2014/),^7^ very limited information is available about these viruses. To better understand the genetic and biologic characteristics of the novel H5N8 virus, we sequenced the entire viral genome, analyzed the receptor-binding properties of the hemagglutinin (HA) protein, and evaluated viral pathogenicity in mice.

## Methods

Wild bird oral and rectal swabs were collected from 14 mallard ducks, which were captured for bird banding in Shanghai city (31°30′N, 121°45′E) in December of 2013. Samples were inoculated into embryonated specific pathogen-free (SPF) chicken eggs for virus isolation. Positive sample was confirmed using RT-PCR for detecting influenza A matrix. Sequences were aligned using dnastar software (http://www.dnastar.com/default.aspx). Phylogenetic analyses were performed in mega5.1 program (http://www.megasoftware.net/). Receptor specificity testing was performed for A/mallard duck/Shanghai/SH-9/2013 (SH-9) using a solid-phase binding assay with α-2,6- and α-2,3-linked glycans as previously reported with minor modifications.^8^ To determine the 50% mouse lethal dose (MLD50) of infected mice, groups of six female 5-week-old BALB/c mice were inoculated intranasally with 50 μl 10-fold serially diluted virus fluid. Twelve female 6-week-old BALB/c mice were inoculated intranasally with 50 μl 10^6·0^EID_50_ SH-9 virus and were monitored for 14 days to determine the body weight changes and mortality. On days 2 and 4 post-inoculation (p.i.), three mice were anesthetized and the nasal turbinates, lungs, brains, livers, kidneys, and colons were collected for virus titration.

## Results

**Figure 1:**
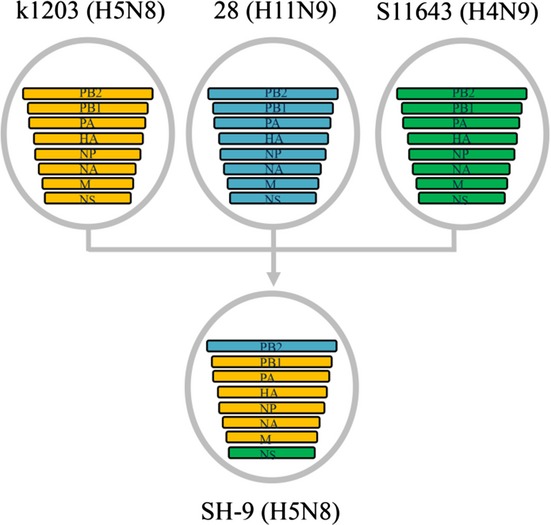
Putative genomic compositions of the novel avian influenza (H5N8) virus with possible donor viruses. The genomic segments of three putative donor viruses are coded by color with each of the eight viral genomic segments represented by a horizontal bar. k1203 (H5N8), A/duck/Jiangsu/k1203/2010; 23(H11N9), A/environment/Jiangxi/28/2009; S11643 (H4N9), A/duck/Hunan/S11643/2013; SH-9 (H5N8), A/mallard duck/Shanghai/SH-9/2013.

An H5N8 virus was isolated from oral and rectal swabs of one mallard duck [A/mallard duck/Shanghai/SH-9/2013 (SH-9)]. We sequenced all eight genomic segments of the novel H5N8 SH-9 virus. BLAST analysis revealed that the HA, NA, PB1, PA, NP, and M segments of SH-9 were most closely related to the corresponding segments of A/duck/Jiangsu/k1203/2010 (H5N8), with each segment displaying 98–99% nucleotide and amino acid sequence identity (Table 1). The SH-9 PB2 segment was most closely related to an H11N9 environmental isolate collected in Jiangxi province, China, and showed 99% nucleotide and amino acid sequence identity (Table 1). The SH-9 NS segment was most closely related to A/duck/Hunan/S11643/2013 (H4N9), with nucleotide and amino acid identities of 99% and 96%, respectively (Table 1). It therefore appeared that the H5N8 virus was a natural recombinant composed of viral gene segments derived from H5N8, H11N9, and H4N9 avian influenza viruses (Figure 1 and Appendix Figure A–H). Recently, three Korea strains (Gochang1, Buan2, and Donglim3) are available in the NCBI,^7^ Phylogenetic analysis shown that Gochang1 falls into same clade with SH-9, with nucleotide identities of 98–99% (Appendix Figure A–H), and these results indicated that strain Gochang1 likely originated from the novel H5N8 SH-9 virus.

**Table 1:** Comparisons of A/mallard/Shanghai/SH-9/2013(H5N8) with isolates in GenBank of highest nucleotide and amino acid identity (%)*

Gene	Site	Nucleotide sequence isolate with the highest homology	Homology (%)	Site	Amino acid sequence isolate with the highest homology	Homology (%)
PB2	14-2293	A/environment/Jiangxi/28/2009(H11N9)	99	1-759	A/environment/Jiangxi/28/2009(H11N9)	99
PB1	25-2298	A/duck/Jiangsu/k1203/2010(H5N8)	99	1-757	A/duck/Jiangsu/k1203/2010(H5N8)	99
PA	25-2175	A/duck/Jiangsu/k1203/2010(H5N8)	99	1-716	A/duck/Eastern China/1111/2011(H5N2)	99
HA	29-1732	A/duck/Jiangsu/k1203/2010(H5N8)	99	1-568	A/duck/Jiangsu/k1203/2010(H5N8)	98
NP	46-1542	A/duck/Jiangsu/k1203/2010(H5N8)	99	1-499	A/goose/Eastern China/1112/2011(H5N2)	99
NA	21-1433	A/duck/Jiangsu/k1203/2010(H5N8)	99	1-471	A/duck/Jiangsu/k1203/2010(H5N8)	99
M	26-1007	A/duck/Jiangsu/k1203/2010(H5N8)	99	1-252	A/duck/Jiangsu/k1203/2010(H5N8)**	99
NS	27-864	A/duck/Hunan/S11643/2012(H4N9)	98	1-230	A/duck/Hunan/S11547/2012(H4N9)***	96

* Comparisons were performed by using the Blast search tool.

** Amino acid sequence of M1 protein was compared.

*** Amino acid sequence of NS1 protein was compared.

Phylogenetic characterization of the SH-9 HA gene placed the novel H5N8 virus in clade 2.3.4.6 (Appendix Figure A). This clade contains recent H5N8 isolates and is located at the extremity of the phylogenetic tree, indicating active virus evolution. The SH-9 HA protein has a series of basic amino acids (REKRRKR/K) at the cleavage site, suggesting that proteolytic activation of the HA protein can be mediated by ubiquitous proteases to enable broad organ tropism and a highly pathogenic phenotype in poultry.^9^ The SH-9 HA possesses a glutamine at amino acid position 226 (H3 numbering), which has been found in the majority of avian H5 isolates and confers binding to avian cell-surface receptors.^10^ The SH-9 PB2 has a glutamic acid residue at position 627 and an aspartic acid residue at position 701, demonstrating that SH-9 did not possess the E627K or D701N substitutions commonly associated with adaptation of avian-derived H5N1 viruses to mammalian hosts.^11^–^13^

**Figure 2:**
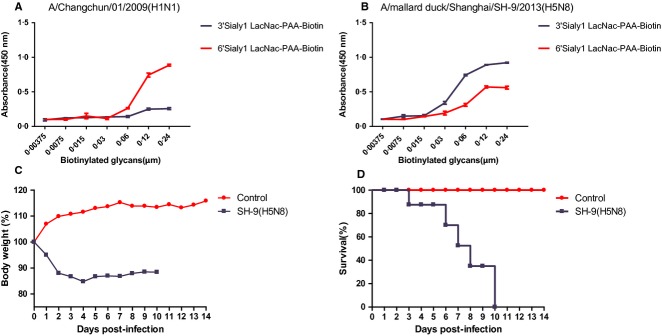
Receptor-binding properties and pathogenicity of the novel H5N8 SH-9 virus in mice. (A, B) Receptor-binding specificities of a human H1N1 virus collecting during the 2009 pandemic (A/Changchun/01/2009(H1N1), panel (A) and the novel SH-9 virus (A/mallard duck/Shanghai/SH-9/2013 (H5N8), panel (B) were evaluated using a biotinylated α-2,3 glycan (blue line) and an α-2,6 glycan (red line). (C, D) Weight loss (C) and survival (D) were monitored for 14 days following inoculation of mice with 10^6·0^ EID_50_ SH-9 virus. Weight loss is expressed as the mean of percent starting body weight for mice at each time point.

HA receptor specificity plays a central role in the host range restriction of influenza viruses. The HA of human influenza viruses preferentially recognize α-2,6-linked sialic acids (human-like receptor), whereas the HA of avian influenza viruses preferentially recognize α-2,3-linked sialic acids (avian-like receptor).^14^ As expected, a human H1N1 virus collecting during the 2009 pandemic (A/Changchun/01/2009) preferentially bound human-like α-2,6-linked sialic acids(*P* < 0·05) (Figure 2A). In contrast, the SH-9 virus displayed a strong preference for avian-like receptors and preferentially bound α-2,3-linked sialic acids (*P* < 0·05) (Figure 2B).

We next investigated the pathogenicity of the novel H5N8 virus in a mouse model. Six-week-old female BALB/c mice were inoculated intranasally with 10^6·0^ EID_50_ SH-9 virus. Mice inoculated with SH-9 exhibited rapid weight loss during the first 2 days post-infection (p.i.), and all mice succumbed to infection by day 10 (Figure 2C, D). SH-9 replicated to high titers in nasal turbinates (10^5·5^ EID_50_/ml) and lungs (10^5·0^ EID_50_/ml) of infected mice by day four p.i. without prior adaptation. In addition, low levels of virus were recovered from the brain (0·98 ± 0·2), livers (1·1 ± 0·3), kidneys (1·3 ± 0·1), and colons (2·3 ± 0·4) of mice on day 4 p.i. The mouse 50% lethal dose (MLD_50_) of SH-9 is 4·8 log_10_EID_50_. These results demonstrate that the SH-9 H5N8 virus replicates systemically and is highly pathogenic in mice.

## Discussion

Shanghai is a desirable site to monitor influenza virus in China, as it is located on the East Asia migratory bird flyway, which spans portions of China, Japan, the Democratic People's Republic of Korea, and the Republic of Korea. Our study suggests that the novel reassortant H5N8 avian influenza virus, which was isolated from an apparently healthy mallard duck, could spread among the East Asia flyway via migratory birds. The recent H5N8 outbreaks in the Republic of Korea support this possibility.^7^

The potential ability of avian influenza viruses to efficiently infect and transmit among human hosts represents a major global public health concern. Mammalian adaptation of avian viruses is associated with a number of molecular markers that confer human-type receptor recognition^8^,^13^ and increase viral replicative capacity in mammals.^11^–^13^ The SH-9 H5N8 HA protein possesses a glutamine at amino acid position 226 which is known to confer binding to avian-like receptors and was shown to preferentially recognize avian-like α-2,3-linked sialic acids in a solid-phase binding assay (Figure 2B). Further, SH-9 has not acquired adaptive changes in polymerase complex proteins, including PB2 627K and 701D, which are associated with increased virulence and viral replication in mammals. Despite the lack of these adaptive changes, the SH-9 virus was capable of systemic replication in mice and resulted in 100% mortality. Mice are a commonly used animal model in which to investigate influenza virus virulence, as H5N1 virus virulence in mice correlates with that seen in humans.^15^

Our study confirms that long-term and systematic active influenza surveillance in important wild bird congregation zones and domestic poultry provides valuable epidemiologic information and enables the detection of emerging viral threats.

## GenBank accession numbers

The genomes reported in this study have been deposited in the GenBank database under accession no. KJ476654 to KJ476661.

## APPENDIX

### Appendix 1: Figures

Phylogenetic characterization of the novel H5N8 SH-9 virus based on the HA, NA, PB2, PB1, PA, NP, M, and NS genes of selected avian influenza viruses. Tree was created using the neighbor-joining method in mega 5.0 (http://www.megasoftware.net) and the coding sequences of the HA segment. Bootstrap values (based on 1000 replicates) for each node are provided when >75%. Horizontal branch length is proportional to genetic distances. The black dot indicates the novel H5N8 virus isolated in this study. (A) Phylogenetic tree generated using HA gene nucleotide sequences. (B) Phylogenetic tree generated using NA gene nucleotide sequences. (C) Phylogenetic tree generated using PB2 gene nucleotide sequences. (D) Phylogenetic tree generated using PB1 gene nucleotide sequences. (E) Phylogenetic tree generated using PA gene nucleotide sequences. (F) Phylogenetic tree generated using NP gene nucleotide sequences. (G) Phylogenetic tree generated using M gene nucleotide sequences. (H) Phylogenetic tree generated using NS gene nucleotide sequences.
